# Dietary Patterns and Retinal Vessel Caliber in the Irish Nun Eye Study

**DOI:** 10.1007/s12603-017-0992-2

**Published:** 2017-12-05

**Authors:** Charlotte E. Neville, S. Montgomery, G. Silvestri, A. McGowan, E. Moore, V. Silvestri, C. Cardwell, C. T. McEvoy, A. P. Maxwell, J. V. Woodside, G. J. McKay

**Affiliations:** 1 Centre for Public Health, School of Medicine, Dentistry and Biomedical Sciences, Queen’s University Belfast, Belfast, UK; 2 Institute for Global Food Security, Queen’s University, Belfast, UK; 3 Centre for Experimental Medicine, Queen’s University Belfast, Belfast, UK; 4 Department of Ophthalmology, Belfast Health & Social Care Trust, Belfast, UK

**Keywords:** Dietary patterns, retinal vessel caliber, cross-sectional

## Abstract

**Background:**

Retinal vessel abnormalities are associated with cardiovascular disease risk. Widening of retinal venules is associated with increased risk of stroke while narrowing of retinal arterioles independently predicts incident hypertension, coronary heart disease and diabetes. Dietary factors are known to play an important role in cardiovascular health. However, few studies have examined the association between dietary patterns (DPs) and retinal microvascular health.

**Objective:**

To examine the association between ‘a posteriori’- derived DPs and retinal vascular caliber (RVC) in older women with a restricted lifestyle.

**Methods:**

This was a cross-sectional study of 1233 participants (mean age: 76.3 years) from the Irish Nun Eye Study (INES). Computer-assisted software was used to measure RVC from digital eye images using standardized protocols. Dietary intake was assessed using a food frequency questionnaire (FFQ). DP analysis was performed using principal component analysis from completed FFQs. Regression models were used to assess associations between DPs and retinal vessel diameters, adjusting for age, body mass index, refraction, hypertension, diabetes mellitus, ischemic heart disease, cerebrovascular accident and fellow eye RVC.

**Results:**

Two DPs were identified: a ‘healthy’ pattern with high factor loadings for fruit, vegetables, wholegrains and oily fish and an ‘unhealthy’ pattern with high factor loadings for sugar and sweets, chips, high fat dairy products and French fries. Adjusted linear regression analysis revealed that those who adhered most closely to the unhealthy DP had wider central retinal venular equivalent (CRVE) (p=0.03) and narrower central retinal arteriolar equivalent (CRAE) (p=0.01) compared to the least unhealthy DP. No independent relationship was observed between the healthy DP and RVC.

**Conclusion:**

In this cohort of older women with a restricted lifestyle, an unhealthy DP was independently associated with an unfavorable retinal profile, namely a widening of retinal venules and narrowing of retinal arterioles.

## Introduction

Evidence from observational epidemiological studies and clinical trials strongly supports a role for the consumption of a healthy diet in the prevention of cardiovascular disease (CVD) (1-4).

The relationship between dietary intake and vascular disease may also be partly mediated by changes in the microcirculatory system (5, 6). Indeed, in recent years there has been increased interest in the use of retinal imaging as a noninvasive means of examining the retinal microvasculature, thus providing an objective means of measuring vascular health and potentially identifying individuals at increased risk of vascular disease. Subtle changes in retinal microvasculature and retinal vessel abnormalities may reflect changes in the wider systemic vasculature. Specifically, widening of retinal venules has been associated with incident CVD while narrowing of retinal arterioles independently predicts incident hypertension, coronary heart disease (CHD) and diabetes mellitus (DM) (7-10). Gender differences have also been confirmed in the association between retinal microvasculature and CHD risk, with wider retinal venules and narrower retinal arterioles being associated with increased risk in women, but not men (11).

Few European based studies have examined the relationship between dietary intake and retinal microvascular health (12, 13). Of the studies conducted in the US, Australia and Singapore most have focused on the association between individual nutrients or foods and microvascular health (5, 14-18) rather than examining the variety of foods consumed in the overall diet. Dietary pattern (DP) analysis offers the ability to capture the complexity and quality of overall dietary intake, thereby incorporating potential interactions that can occur among different foods and nutrients (19, 20). A commonly used approach to assess DPs is the ‘a posteriori’ method. ‘A posteriori’ patterns are empirically derived from dietary data using exploratory multivariate statistical techniques such as principal component analysis (PCA), which typically aggregates foods that are frequently consumed together and generates distinctive patterns of food consumption for the study population. To our knowledge, only two studies to date have investigated the association between ‘a posteriori’ DPs and retinal microvascular health, one study showed no association between DPs and retinal vessel caliber (RVC) (13) while the other study reported that a DP characterized by low fish and vegetable intake was associated with an adverse retinal venular caliber (21). However, it is difficult to compare studies as the former study was conducted in 288 older adults while the latter study was based on a small sample of 83 children and adolescents with type 1 DM. Therefore, the aim of this study was to examine the association between ‘a posteriori’-derived DPs and RVC in a highly specific cohort of older women with relatively stable and restricted lifestyle behaviors.

## Methods

### Study population

DP data were collected as part of the Irish Nun Eye Study (INES), a cross-sectional study which primarily examined the prevalence of age-related macular degeneration (AMD) in nuns, a population with a restricted lifestyle, and risk factors associated with AMD. A restricted lifestyle or monastic rule is a set of restrictions that governs behavior in terms of material possessions, emotional and physical attachment, maintenance of a daily structured religious life of abstinence and prayer with dietary and lifestyle limitations. Sampling procedures and study design have been described previously (22, 23). In brief, the study was conducted between 2007 and 2009 in 1233 nuns recruited from 126 convents across Ireland. All participants were of Irish descent, white ethnicity, aged over 55 years and had lived in a convent for 25 years or more. Written informed consent was obtained from participants prior to participation and ethical approval obtained from the Office for Research Ethics Committee Northern Ireland. For the purposes of this study, the DP analyses were based on 1033 participants who completed a food frequency questionnaire (FFQ) as part of the study. Of these 1033 participants, 941 also had retinal images available. Images were not available for 92 participants due to difficulties with image acquisition resulting in insufficient quality of the images for vessel assessment. The difficulties in image acquisition occurred as a result of postural complications with the elderly participant, poor pupillary dilation, the presence of an artificial eye or an out of focus image.

### Assessment of dietary intake

Dietary intake was assessed using the validated, semiquantitative Scottish Collaborative Group (SCQ) FFQ (http://www.foodfrequency.org/) consisting of 170 food items grouped into 19 sections. For each food item, participants specified their frequency of consumption over the previous 2–3 months in terms of measures/day (1, 2, 3, 4, 5+) (equivalent to a standard portion) and days/week (1, 2, 3, 4, 5, 6, or 7 days/week, more than once a month, rarely). Foods reported were converted to a daily weight using food portion sizes (24).

### Dietary pattern identification

The food items in the FFQ were manually grouped into 38 food groups based on macronutrient content and food type (Table 1). DPs were generated from these food groups using PCA with orthogonal (varimax) rotation. The PCA generated factor loadings for each food group. The number of factors retained was determined from the scree plot and based on the number of factors above the breakpoint. Food groups with factor loadings greater than 0.2 were retained to help interpretation. For each DP, the PCA produced scores for each participant, which were subsequently categorized into fifths. The fifth category indicated greatest conformity to that particular DP.

### Assessment of RVC and fractal dimension

Retinal arteriolar and venular calibers were measured from optic disc-centered digital retinal images using Interactive Vessel ANalysis software (IVAN; University of Wisconsin, Madison, WI) as previously described (11). Fractal analysis was performed using the semi-automated Singapore I Vessel Assessment-Fractal Analyzer (SIVA-FA, software version 1.0, School of Computing, National University Singapore) according to a standardized protocol (25). Measurements were performed by a single trained grader (AMcG), blinded to the participants’ characteristics. Images were taken from the right eye except when unavailable, in which case the left eye image was used. Reproducibility of the retinal vascular measurements has been reported previously (23). A medical questionnaire was used to assess medical and ocular history. Refractive error was recorded from a recent prescription or from the participants’ glasses. Where glasses were not available, corrected visual acuity was achieved by pinhole correction: refraction was not carried out.

### Demographic, lifestyle and anthropometric data

A structured questionnaire was used to assess smoking and alcohol status, medication usage and disease status (presence or absence). Systolic (SBP) and diastolic blood pressure (DBP) was measured in a seated position using an oscillometric blood pressure aneroid sphygmomanometer (Speider and Keller). Mean arterial blood pressure was calculated as one third of SBP plus two thirds of DBP. Height and weight were measured and body mass index (BMI) computed as weight (kilograms)/height (meters) squared.

### Statistical analysis

Descriptive statistics were obtained for all variables of interest. Continuous and categorical variables were summarized as mean (SD) and n (%) respectively. Measurements of RVC were summarized as CRAE (Central Retinal Arteriolar Equivalent) and CRVE (Central Retinal Venular Equivalent) according to the revised Knudston-Hubbard formula (26) which represents the average caliber of the arterioles and venules in each eye examined. Quantitative RVC (CRAE and CRVE) and fractal dimension was assessed as a continuous variable. One-way analysis of variance and multivariable linear regression analyses were used to examine the relationship between the identified DPs (with the distribution of each DP divided into equal fifths) and mean CRAE, CRVE and fractal dimension in both unadjusted (Model 1) and adjusted analyses (Models 2, 3 and 4). The lowest fifth of each DP, reflecting lowest adherence to the pattern, was used as the reference category. Confounding factors included in the analyses were those identified from similar studies in the literature and those known to influence vascular health (13, 22, 26). Model 1 was unadjusted; Model 2 was adjusted for refraction, age, BMI; Model 3 was adjusted as for Model 2 plus ever smoker, alcohol status, hypertension, DM, ischemic heart disease (IHD) and cerebrovascular accident (CVA); Model 4 was adjusted as for Model 3 plus fellow vessel caliber (i.e. CRAE as a covariate in the analysis of CRVE and vice versa) as suggested previously (27). Statistical analyses were performed using SPSS version 21 (SPSS Inc., Chicago, IL). For all analyses, p<0.05 was considered statistically significant.

**Table 1:**
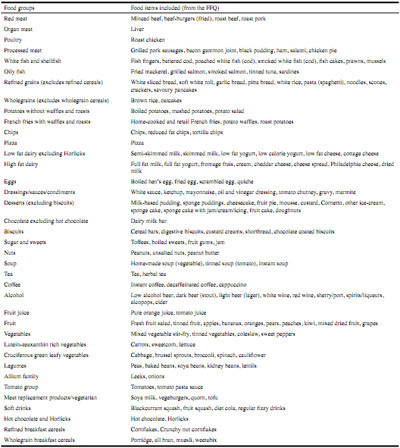
Food groupings used in the dietary pattern analysis

## Results

The characteristics of female participants are shown in Table 2. The mean age of the sample was 76.3 years. Based on BMI classifications, 12% (n=111) of participants were defined as underweight (i.e. BMI <18.5 kg/m2), 45% (n=412) were normal weight (i.e. BMI 18.5–25 kg/m2), 28% (n=258) were overweight (i.e. BMI 25–30 kg/m2) and 15% (n=138) were clinically obese (i.e. BMI >30 kg/m2). The majority (96%) had never smoked and were non-consumers of alcohol. Approximately 3% of participants had self-reported CVA, 11% had IHD and 3% had DM. Mean (SD) systolic and diastolic blood pressure was 131.6 (17.5) and 72.9 (9.4) mmHg, respectively. Hypertension was reported in 41% of participants. Mean (SD) CRAE and CRVE was 120.5 (12.7) and 169.2 μm (18.4), respectively while mean (SD) AVR was 0.72 (0.06).

PCA resulted in two major DPs being derived which were labelled ‘healthy’ and ‘unhealthy’. The factor loadings for the derived DPs are presented in Table 3. The healthy DP was characterized (in decreasing order of factor loadings) by lutein/zeaxanthin-rich vegetables, green leafy vegetables, alliums, vegetables, fruit, tomatoes, legumes, nuts, oily fish, low fat dairy products, pizza, dressings/sauces/condiments, wholegrain breakfast cereal and red meat. The unhealthy DP was characterized (in decreasing order of factor loadings) by chips, French fries, alcohol, high fat dairy products, soups, desserts, sugars and sweets, wholegrains, dressings/sauces/condiments, processed meat, potatoes, eggs, refined grains, refined breakfast cereal, chocolate, vegetables, red meat, white fish and shellfish. Together, these DPs accounted for 16% of the total variance in this population.

Participant characteristics were compared across the fifths of the DPs. No significant differences were observed for the healthy DP except for age (p<0.001) and BMI (p=0.03) where women in the lowest fifth tended to be older and have a higher BMI than those in the other fifths. In the unhealthy DP, participants in the lowest fifth also tended to be older (p=0.01) while those in the higher fifth of the unhealthy DP reported greater alcohol consumption and increased rate of smoking (p<0.001). DM was more prevalent in fifth 2 of the unhealthy DP compared to the other fifths (p=0.01) (data not shown).

**Table 2:**
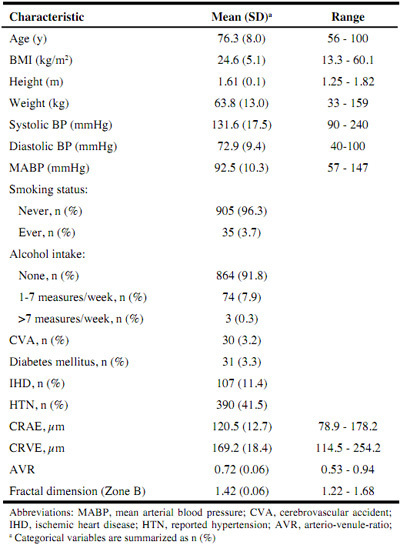
Health and lifestyle characteristics of the Irish Nun Eye Study participants (n_max_=941)

Table 4 shows the difference in retinal vessel parameters across the fifths of each DP. In the healthy DP, a significant difference in CRVE and CRAE was observed between those in the lowest fifth (least healthy) and those in the highest fifth (most healthy). Participants with highest adherence to the healthy DP had on average 3.84 *μ*m (95% CI -7.53, -0.15) narrower CRVE and 2.64 *μ*m (95% CI -5.18, -0.10) narrower CRAE than those with lowest adherence, after adjusting for refraction, age, BMI, smoking and alcohol status, hypertension, DM, IHD and CVA (CRVE p for trend = 0.03; CRAE p for trend = 0.008) (Model 3). However, significance was lost following adjustment for fellow vessel caliber (Model 4, healthy DP).

**Table 3:**
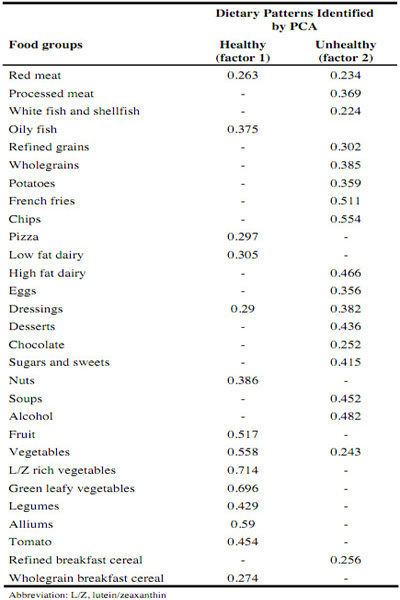
Factor loading matrix for the two major dietary patterns identified in the Irish Nun Eye Study (n=1033)

In the unhealthy DP, a significant difference in CRVE and CRAE was observed between those in the lowest fifth (least unhealthy) and those in the highest fifth (most unhealthy). Participants who adhered most to an unhealthy DP (fifth 5) had on average 2.70 *μ*m (95% CI -0.09, 5.48) wider CRVE and 1.32 *μ*m (-3.24, 0.60) narrower CRAE than those in the lowest fifth, after adjusting for confounding factors. A linear trend was observed across the fifths of the unhealthy pattern for both CRVE (p=0.03) and CRAE (p=0.01) (Model 4, unhealthy DP).

There was no significant difference in the AVR across fifths of the healthy DP, however, those adhering most to the unhealthy DP (fifth 5) had a significantly lower AVR than those in the lowest fifth, after adjusting for confounding factors (p for trend=0.01) (Model 3).

There was no significant difference in fractal dimension (zone B) measurements across fifths of both DPs (data not shown). Additional adjustment for use of medication (any use *vs* none) (diuretics, nitrates, calcium channel blockers, angiotensin converting enzyme inhibitors, beta-blockers, nonsteroidal anti-inflammatory drugs, aspirin, corticosteroids, statins) did not alter the results (data not shown).

## Discussion

In the current population, we identified two major DPs, which were named the “Healthy” and “Unhealthy” patterns. Closer adherence to an unhealthy DP was associated with an unfavourable retinal profile i.e. narrower arterioles and wider venules. Previous reported associations between retinal arteriolar narrowing and hypertension have suggested elevated blood pressure triggers an auto-regulatory response that manifests in increased arteriolar tone, pre-capillary arteriolar narrowing and increased peripheral vascular resistance (23, 28). Our analyses adjusted for the effects of hypertension, suggesting the associations observed were independent of hypertension status. No independent association was evident between a healthy DP and RVC following adjustment for potential confounders.

There is limited evidence regarding the association between DPs and retinal vascular health and no previous studies have been conducted specifically in older women, who are at greater risk of microvascular disease. Only two other studies have specifically examined the relationship between ‘a posteriori’ PCA-derived DPs, and RVC (13, 21). McEvoy et al (13) identified similar DPs to those derived in our study but, unlike our results, found no evidence of an association with RVC in 288 older men and women. Another recent study by Keel et al (21) reported that a DP characterized by low fish and vegetable intake was associated with wider CRVE. This study however was conducted in 83 children and adolescents with type 1 diabetes. Our findings also lend support to those of Gopinath et al (29) who, instead of examining ‘a posteriori’ derived DPs, examined the association between ‘a priori’ derived DPs and RVC. Their study reported that consumption of a highquality diet which closely complied with dietary guidelines was associated with a more favorable retinal vessel profile in terms of wider retinal arterioles and narrower retinal venules.

The current findings somewhat support those of other studies which examined the association between specific foods or nutrients and RVC. Foods that were dominant components of the unhealthy DP in our study were French fries, chips, desserts, sweets, sugar, high fat dairy products and alcohol. Many of these are carbohydrate-rich foods which have previously been associated with narrowing of retinal arterioles and widening of retinal venules (14). Our findings also lend support to those of Li et al (17) who reported a positive association between low diet quality and widening of retinal venules. There is also strong supporting evidence showing an adverse association between these types of foods and overall vascular health (1).

**Table 4:**
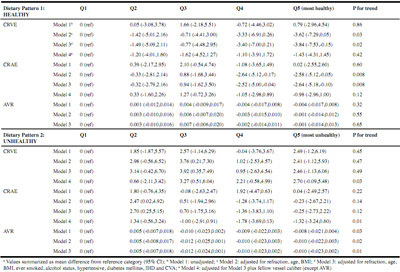
Differences in unadjusted and adjusted RVC across increasing quintile groups of two dietary patterns in participants of the Irish Nun Eye Study^a^

In contrast, fruit and vegetables, oily fish, nuts and low fat dairy products were the foods which dominantly featured in the ‘healthy’ DP within our study. Fruit and vegetables are rich in dietary fiber, which in a previous study (5) were found to be associated with a more favorable retinal vessel profile in terms of narrower retinal venules and wider arterioles. Another study by Kaushik et al (18) reported a positive association between fish consumption, particularly oily fish, and narrowing of retinal venules and widening of arterioles. More recently, Gopinath et al (16) similarly noted that consumption of at least 2 servings of fish per week and omega-3 polyunsaturated fatty acids was associated with wider retinal arterioles in adolescent girls. Consumption of low fat dairy products have also been reported to be beneficial for retinal microvascular health (12, 15). In a cross-sectional study of men and women at increased cardiovascular risk (12), a positive association was observed between low fat dairy products and CRAE and an inverse association with CRVE. These findings show consistency with previous studies which have explored the association between specific foods and vascular health in general. High vegetable intake has been shown to have a vasoprotective effect (30) and is associated with reduced risk of stroke and CHD (31). Omega- 3 fatty acids, which are abundant within oily fish and nuts, have also been shown to confer systemic benefits and vasoprotective effects and have consistently been associated with reduced risk of CVD (18, 32, 33).

Strengths of this study include the large sample size, being one of the largest European-based studies to examine diet and retinal microvasculature in a novel well-characterized older aged cohort. Standardized methods were used to collect data on a wide range of potential confounders and the relative uniformity/homogeneity of the nun’s lifestyles is likely to have reduced potential confounding. A further strength is the high number of gradable digital retinal images that were analyzed by a single blinded trained grader using semi-automated computer-based techniques. Unlike other studies which examined the association between single foods or nutrients and retinal microvascular health, our study used a more novel approach for assessing dietary intake. Examining DPs is a relatively new approach in nutritional epidemiology but is increasingly being used to provide a better understanding of the synergistic interactions between particular foods and the overall pattern of food consumption. Foods and nutrients are rarely eaten in isolation; therefore, the holistic nature of DP analyses reduces residual confounding and enables the overall contribution of different foods to each DP to be explored, rather than focusing on the direct effect of a single nutrient or food. ‘A posteriori’ DPs are unique to each population, and are not limited by predetermined hypotheses linking dietary intake and health. While DPs may vary according to gender, socioeconomic status, ethnic group, and culture, the DPs we identified reflect those identified in previous studies, and are potentially applicable to other populations.

Like all observational studies, this study has limitations. The cross-sectional study design limits its strength in determining causality. It is also possible that the associations observed between an unhealthy DP and retinal measurements simply reflect other underlying lifestyle factors which were not identified or measured. While we adjusted the regression models for health conditions such as diabetes mellitus, IHD and CVA, there may potentially have been other health conditions present which were not accounted for and which may have resulted in recent changes in eating/dietary habits. Blood glucose levels, insulin sensitivity, lipids, CRP and measures of micronutrient status were not available for this cohort. However, the homogeneous lifestyle of this population is likely to have reduced residual confounding. The dietary intake assessment method also has limitations. While the FFQ is widely used in epidemiological studies to capture dietary intake and DPs, it is limited in its ability to assess all dietary components. Although 170 food items were included in the FFQ, it is possible that some other commonly consumed foods were not captured within the FFQ. A further weakness of the FFQ is that, for some foods, the frequency of consumption could not be determined. The cross-sectional study design also meant that dietary intake and retinal imaging was only assessed at one time-point and therefore does not reflect changes over time. Other studies however that used similar methods have shown reasonable reproducibility of dietary patterns over time (34, 35).

The use of PCA to derive DPs also has inherent limitations, the majority of which have been noted previously (13, 21). While PCA is increasingly used in nutritional epidemiology to empirically derive DPs and has shown good reproducibility and validity (36), it is a subjective measure which involves several arbitrary decisions, including the choice and number of food groups, the method of rotation, the number of factors to retain and the naming of the resultant DPs (37). In our study, the DPs identified only accounted for 16% of the total variance in DPs. However, previous studies conducted in white populations have reported similar levels of variance (35, 38, 39).

Overall, this study provided an opportunity to examine the association between DPs and retinal vessels in a highly characterized and ageing cohort. The findings provide further support to the role of diet in microvascular health. In this cohort, an unhealthy DP was independently associated with an unfavourable retinal profile, namely a widening of retinal venules and narrowing of retinal arterioles. Further prospective studies are required in other population groups to confirm the current findings and intervention studies conducted to determine whether change in dietary behavior over time would result in changes in RVC.

*Acknowledgments:* The authors wish to thank all study participants and also Janet Kyle for provision of the food frequency questionnaire and providing advice regarding the set up and design of the study.

*Conflict of Interest:* The authors declare no conflicts of interest with the exception of G.Silvestri who reports grants from NIHPSS R&D during the conduct of the study, personal fees from Bayer and non financial support from Allergan outside the submitted work.

*Ethical standard:* Ethical approval for the study was obtained from the Office for Research Ethics Committee Northern Ireland.
